# The Change of Systemic Immune-Inflammation Index Independently Predicts Survival of Colorectal Cancer Patients after Curative Resection

**DOI:** 10.1155/2020/4105809

**Published:** 2020-11-12

**Authors:** Qingqing Chen, Haohao Wu, Xinwei Guo, Ke Gu, Wenjie Wang, Xiaochen Chen, Shengjun Ji, Hui Yang, Jiahao Zhu

**Affiliations:** ^1^Department of Radiotherapy & Oncology, Nanjing Medical University Affiliated Suzhou Hospital, Suzhou, China; ^2^Department of Radiotherapy, The Yancheng No. 1 People's Hospital, Yancheng, China; ^3^Department of Oncology, Affiliated Taixing People's Hospital of Yangzhou University, Taixing, China; ^4^Department of Radiotherapy & Oncology, Affiliated Hospital of Jiangnan University, Wuxi, China

## Abstract

**Background:**

The systemic immune-inflammation index (SII) has an important role in predicting survival in some solid tumors. However, little information is available concerning the change of the SII (∆SII) in colorectal cancer (CRC) after curative resection. This study was designed to evaluate the role of ∆SII in CRC patients who received surgery.

**Methods:**

A total 206 patients were enrolled in this study. Clinicopathologic characteristics and survival were assessed. The relationships between overall survival (OS), disease-free survival (DFS), and ∆SII were analyzed with both univariate Kaplan-Meier and multivariate Cox regression methods.

**Results:**

Based on the patient data, the receiver operating characteristic (ROC) optimal cutoff value of ∆SII was 127.7 for OS prediction. The 3-year and 5-year OS rates, respectively, were 60.4% and 36.7% in the high-∆SII group (>127.7) and 87.6% and 79.8% in the low-∆SII group (≤127.7). The 3-year and 5-year DFS rates, respectively, were 54.1% and 34.1% in the high-∆SII group and 80.3% and 78.5% in the low-∆SII group. In the univariate analysis, smoking, pathological stages III-IV, high-middle degree of differentiation, lymphatic invasion, vascular invasion, and the high-*Δ*SII group were associated with poor OS. Adjuvant therapy, pathological stages III-IV, vascular invasion, and *Δ*SII were able to predict DFS. Multivariate analysis revealed that pathological stages III-IV (HR = 0.442, 95% CI = 0.236-0.827, *p* = 0.011), vascular invasion (HR = 2.182, 95% CI = 1.243-3.829, *p* = 0.007), and the high-*Δ*SII group (HR = 4.301, 95% CI = 2.517-7.350, *p* < 0.001) were independent predictors for OS. Adjuvant therapy (HR = 0.415, 95% CI = 0.250-0.687, *p* = 0.001), vascular invasion (HR = 3.305, 95% CI = 1.944-5.620, *p* < 0.001), and the high-*Δ*SII group (HR = 4.924, 95% CI = 2.992-8.102, *p* < 0.001) were significant prognostic factors for DFS.

**Conclusions:**

The present study demonstrated that ∆SII was associated with the clinical outcome in CRC patients undergoing curative resection, supporting the role of ∆SII as a prognostic biomarker.

## 1. Introduction

Colorectal cancer (CRC) is one of the most common malignant diseases [1, 2]. The clinical outcome of CRC is still unsatisfactory because of recurrence or metastasis. In addition, despite significant advances in CRC treatment with intense research activity and outcomes, the appropriate stratification of CRC patients remains a challenge. There is still heterogeneity in the prognosis between CRC with the same tumor nodal metastasis (TNM) stage and CRC with the same Dukes' classification [3, 4], which indicates that they are not sufficient to correctly stratify patients in terms of the risk of mortality [5, 6]. Other conventional prognostic biomarkers, such as tumor differentiation and pathological type, have been used for predicting CRC outcomes [7, 8]. However, these are tumor tissue dependent and the detections are usually costly and time-consuming. Therefore, it is very urgent to develop auxiliary biomarkers to help clinicians to identify individualized treatment.

In recent years, a lot of inflammation-based prognostic systems are gradually getting attention [9, 10], with the role of inflammation in angiogenesis promotion, DNA damage, and tumor invasion and metastasis [11–13]. Several immune-inflammation systems, such as the neutrophil-lymphocyte ratio (NLR), platelet-lymphocyte ratio (PLR), and lymphocyte-to-monocyte ratio (LMR), have been reported to predict the prognosis for some malignant solid tumors [14–17]. Systemic immune-inflammation index (SII) is a novel parameter related to the platelet, neutrophil, and lymphocyte. Recently, SII has been reported for its prognostic role in some solid tumors such as liver cancer, non-small-cell lung cancer, renal cell cancer, and esophageal squamous cell carcinoma [18–20]. Although many studies also indicated the prognostic role of SII in CRC patients, the results were inconsistent [21–23]. Noteworthy, some studies have found that the dynamic change in the SII represents a new indicator for predicting the prognosis in renal cell cancer [20] and hepatocellular carcinoma [24]. However, the role of the change in SII (∆SII) before and after treatment is unknown for patients undergoing curative surgery for CRC. Therefore, we designed the study to evaluate the prognostic value of ∆SII in CRC patients who received curative resection.

## 2. Materials and Methods

### 2.1. Patients

In total, 206 sequential patients diagnosed with CRC who were treated at our institute from February 2010 to May 2015 were collected. The tumor stage was classified according to the seventh edition of the American Joint Committee on Cancer (AJCC) TNM classification system. The clinicopathological characteristics and laboratory data were collected for each patient. Patients were included if they underwent surgical resection and had sufficient laboratory data and follow-up data available. Patients with infection and emergency cases or patients using anti-inflammatory or immunosuppressive medicines were excluded. We also excluded the patients who received neoadjuvant therapy, chemotherapy, or radiotherapy. Follow-up assessments included routine laboratory and physical examinations as well as imaging examinations every 3 months in the first 3 years and every 6 months thereafter. The end points were overall survival (OS) and disease-free survival (DFS).

### 2.2. Ethical Statement

All procedures performed were in accordance with the ethical standards of the responsible committee for human experimentation (institutional and national) and with the Helsinki Declaration of 1964 and later versions. This study was approved by the Institutional Review Board of Nanjing Medical University Affiliated Suzhou Hospital (No. KL901056).

### 2.3. Calculation and Definition of SII and *Δ*SII

The platelet, neutrophil, and lymphocyte count at 7-day preoperative and postoperative. The SII was calculated as follows: SII = plateletcounts∗neutrophilcounts/lymphocytecounts. The *Δ*SII (change in SII) was calculated as the postoperative SII minus the preoperative SII.

### 2.4. Statistical Analysis

Association analysis was performed with the Fisher exact test or the chi-squared test, when appropriate. The Kaplan-Meier method and log-rank test were used for subsistence analysis. OS was the time between surgery and death or the last follow-up. DFS was the time between surgery and the first relapse (local recurrence and/or distant metastases). Univariate and multivariate Cox regression analyses were used to evaluate independent prognostic value. The optimal cutoff value was calculated with receiver operating characteristic (ROC) curves. All tests were two-sided, and *p* < 0.05 was considered to be significant. The SPSS 25.0 statistical software (IBM Corporation, Armonk, NY, USA) was used to statistical analysis.

## 3. Results

### 3.1. Patient Characteristics

A total of 206 CRC patients received surgical were enrolled in the study. The median age was 56.8 years (27-83 years). The median follow-up duration was 46.5 months (8-98 months). The male and female patients were 108 (52.4%) and 98 (47.6%), respectively. In total, the number of early-stage (I-II) patients was 109 (52.9%) and the number of advanced-stage (III-IV) patients was 97 (47.1%). A total of 101 (49.0%) patients received adjuvant therapy (radiation, chemotherapy, or radiochemotherapy). The baseline of patient characteristics is shown in Table 1.

### 3.2. Survival Results and Prognostic Values

For all patients, before the last follow-up, 64 (31.1%) patients died, and 74 (35.9%) patients developed recurrence. The median DFS and OS were 44.6 months (2-80 months) and 51.6 months (8-98 months), respectively. The 3-year and 5-year OS rates were 80.1% and 34%. The 3-year and 5-year DFS rates were 71.4% and 25.7% for the entire study population.

### 3.3. Overall Survival Prediction with ROC Curve

**Figure 1:**
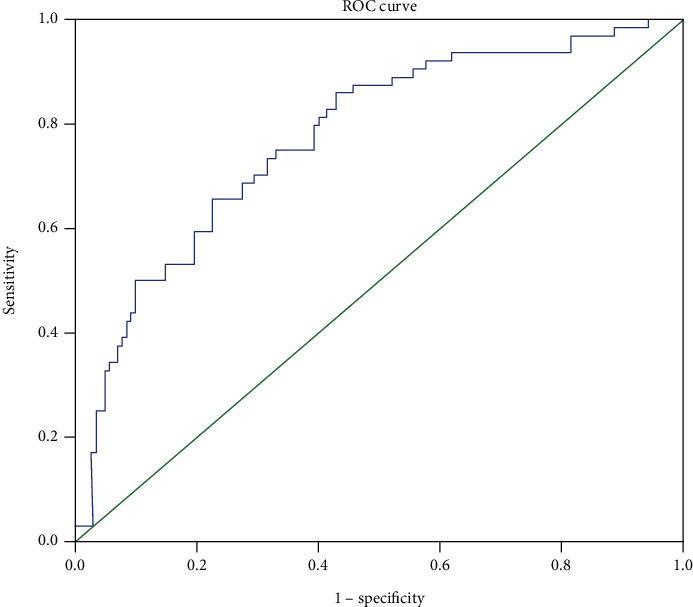
Receiver-operating characteristic curves for overall survival prediction.

We attempted to select the optimal cutoff for the *Δ*SII in our study with ROC curve analysis. The optimal cutoff value for the ∆SII was127.7 for the OS prediction, with an AUC of 0.774 (sensitivity = 65.6% and specificity = 77.5%) (Figure 1). Consequently, the patients were divided into two groups, the high ∆SII group (ΔSII > 127.7) or low ∆SII group (ΔSII ≤ 127.7). 74 patients (35.9%) with high ∆SII were considered the high-risk group, and 132 patients (64.1%) with low ∆SII were considered the low-risk group.

### 3.4. The *Δ*SII and Clinicopathological Characteristics

The clinicopathological characteristics in the high-*Δ*SII group (>127.7) and low-*Δ*SII group (≤127.7) are shown in Table 2. There were no significant differences between the two groups, except for age (*p* < 0.001), adjuvant therapy (*p* = 0.016), and vascular invasion (*p* = 0.013).

### 3.5. Overall Survival and Disease-Free Survival according to the *Δ*SII

**Figure 2:**
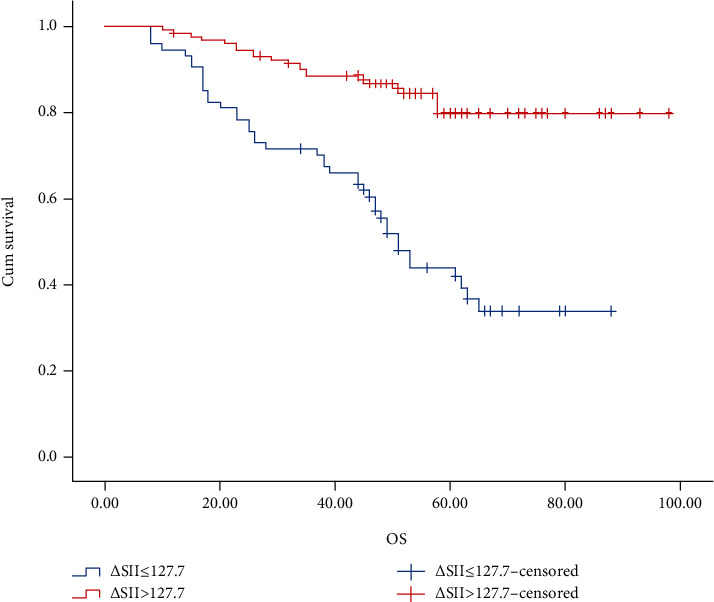
Kaplan-Meier graphs of overall survival for patients in the high-*Δ*SII group (>127.7) and low-*Δ*SII group (≤127.7).

**Figure 3:**
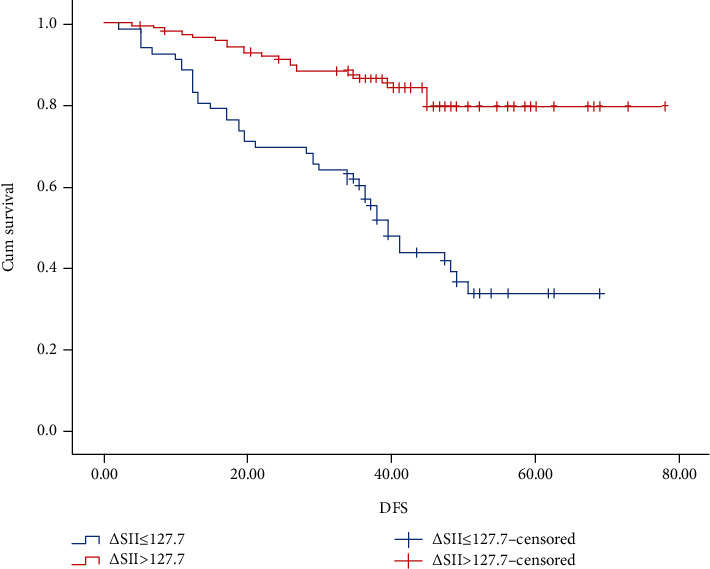
Kaplan-Meier graphs of disease-free survival for patients in the high-*Δ*SII group (>127.7) and low-*Δ*SII group (≤127.7).

The OS and DFS was estimated with the Kaplan-Meier method (Figures 2 and 3). The 3-year and 5-year OS rates, respectively, were 60.4% and 36.7% in the high-∆SII group and 87.6% and 79.8% in the low-∆SII group. The 3-year and 5-year DFS rates, respectively, were 54.1% and 34.1% in the high-∆SII group and 80.3% and 78.5% in the low-∆SII group.

### 3.6. Univariate and Multivariate Cox Regression Survival Analyses

In the univariate analysis, smoking (HR = 1.890, 95% CI = 1.028-3.476, *p* = 0.040), pathological stages III-IV (HR = 0.451, 95% CI = 0.270-0.755, *p* = 0.002), a high-middle degree of differentiation (HR = 0.476, 95% CI = 0.262-0.862, *p* = 0.014), lymphatic invasion (HR = 1.934, 95% CI = 1.159-3.227, p =0.012), vascular invasion (HR = 3.508, 95% CI = 1.771-5.279, *p* < 0.001), and the *Δ*SII (HR = 4.281, 95% CI = 2.553-7.719, *p* < 0.001) were associated with poor OS (Table 3). Adjuvant therapy (HR = 0.627, 95% CI = 0.395-0.993, *p* = 0.047), pathological stages III-IV (HR = 0.512, 95% CI = 0.321-0.814, *p* = 0.005), vascular invasion (HR = 3.572, 95% CI = 2.118-6.024, p<0.001), and the *Δ*SII (HR = 4.041, 95% CI =2.514-6.496, p<0.001) were able to predict DFS (Table 4). The multivariate analysis revealed that pathological stages III-IV (HR = 0.442, 95% CI = 0.236-0.827, *p* = 0.011), vascular invasion (HR = 2.182, 95% CI = 1.243-3.829, *p* = 0.007), and the *Δ*SII (HR =4.301, 95% CI =2.517-7.350, p<0.001) were independent predictors of OS (Table 3). Adjuvant therapy (HR = 0.415, 95% CI = 0.250-0.687, *p* = 0.001), vascular invasion (HR = 3.305, 95% CI = 1.944-5.620, *p* < 0.001), and the *Δ*SII (HR = 4.924, 95% CI = 2.992-8.102, *p* < 0.001) were significant prognostic factors for DFS (Table 4).

## 4. Discussion

In this study, we investigated the association between the ∆SII and the clinical outcome of CRC. Our study demonstrated that the ∆SII had an independent prognostic value in CRC patients who underwent curative resection. Our study suggested that the ∆SII could independently predict survival in colorectal cancer after curative resection.

Some inflammatory index, such as the NLR, LMR, and PLR, have been indicated to be valid prognosticators for CRC [17, 25–27]. However, these prognosticators are typically based on two immunoinflammation cell types, and their predictive reliability for clinical outcomes was limited. The SII has been widely investigated and was demonstrated to be an effective predictor of the prognosis of various malignant tumors [18–20, 28, 29]. Hu et al. [19] initially revealed the prognostic value of the SII in liver cancer and demonstrated that higher preoperative SII heralded shorter survival times. Tong et al. [28] also demonstrated that a high SII was associated with poor outcomes in non-small-cell lung cancer. Jiang et al. [29] conducted a propensity score-matched analysis, which showed that the SII could predict OS in nasopharyngeal carcinoma patients independently.

However, most previous studies only indicated the preoperative value and the results were not consistent. Some studies indicate that SII was proposed as a significant prognostic factor in CRC [21, 22, 30], whereas other studies were not significant [23, 31]. Few studies focused on the changes of inflammatory markers before and after treatment, which might reflect the correlation between the host's inherent inflammatory state and immune response. Wang et al. [24] reported that dynamic changes in the SII represent new prognosis indicators for liver cancer that received surgery. As far as we know, this present study was the first to explore the role of the ∆SII in CRC. These results revealed that the ∆SII (*p* < 0.001) was an independent risk factor that predicted OS. Furthermore, we confirmed the significant predictive value of the ∆SII (*p* < 0.001) for DFS. We also observed that the surgical approach, CEA level, differentiation degree, and lymphatic invasion were not significantly associated with OS and DFS for CRC patients. Adjuvant therapy was only associated with DFS. These results revealed that the ∆SII reflects the systemic immune-inflammatory changes before and after surgery, which may indicate a more accurate prognosis.

The underlying mechanism by which ∆SII predicts the survival of CRC may be related to platelets, neutrophils, and lymphocytes. Elevated platelet counts can protect circulating tumor cells (CTCs) and promote metastasis by inducing CTC epithelial-mesenchymal transition [32, 33]. Neutrophils have been reported to have the potential to promote tumor progression by establishing a tumor microenvironment, including a lot of inflammatory index, such as growth factors (CXCL8), proangiogenic factors (VEGF), and antiapoptotic factors (NF-*κ*B) [34–36]. Lymphocytes can inhibit tumor cell proliferation and migration by secreting cytokines, inducing cytotoxic cell death [37]. Therefore, a higher SII indicates a stronger inflammatory reaction while weaker immune defense, leading to poor clinical outcomes.

There are several limitations of this study. Firstly, the current study used a retrospective design with a small population. Although we utilized data that was obtained from the electronic medical records, biases such as selection bias may exist. Secondly, the impact of treatment after recurrence on survival has not been analyzed due to lack of sufficient information. Therefore, further studies with larger sample sizes and prospective research designs are necessary to demonstrate the relationship between the ∆SII and prognosis for CRC patients.

In conclusion, our study demonstrated ∆SII was an independent prognostic factor for CRC patients undergoing curative resection. The patients with CRC would receive more individualized treatment according to the ∆SII in the future.

## Figures and Tables

**Table 1:** Baseline characteristics.

Variables	*n*	%
Age (years)		
>60	73	35.4
≤60	133	64.6
Gender		
Male	108	52.4
Female	98	47.6
Smoking status		
Yes	63	30.6
No	143	69.4
^†^KPS		
>80	148	71.8
≤80	58	28.2
Localization		
Rectum	121	58.7
Colon	85	41.3
Surgical approach		
Laparoscopy	117	56.8
Open	89	43.2
Adjuvant therapy		
Yes	101	49.0
No	105	51.0
^†^CEA (ng/ml)		
>5	89	43.2
≤5	117	56.8
Pathological stage		
Stages I–II	109	52.9
Stages III–IV	97	47.1
Differentiated degree		
High-middle	173	84.0
Minor	33	16.0
Lymphatic invasion		
Yes	99	48.1
No	107	51.9
Vascular invasion		
Yes	99	48.1
No	107	51.9

^†^KPS: Karnofsky Performance Status; CEA: carcinoembryonic antigen.

**Table 2:** Clinicopathological variables of 206 colorectal cancer patients according to the *Δ*SII.

Variables	^†^ΔSII > 127.7	ΔSII ≤ 127.7	*p*
	*N* = 74 (35.9%)	*N* = 132 (64.1%)	
Age (years)			
>60	38 (51.4)	35 (26.5)	<0.001
≤60	36 (48.6)	97 (73.5)	
Gender			
Male	33 (44.6)	75 (56.8)	0.092
Female	41 (55.4)	57 (43.2)	
Smoking status			
Yes	20 (27.0)	43 (32.6)	0.407
No	54 (73.0)	89 (67.4)	
^†^KPS			
>80	51 (68.9)	97 (73.5)	0.485
≤80	23 (31.1)	35 (26.5)	
Localization			
Rectum	46 (62.2)	75 (56.8)	0.455
Colon	28 (37.8)	57 (43.2)	
Surgical approach			
Laparoscopy	42 (56.8)	75 (56.8)	0.993
Open	32 (43.2)	57 (43.2)	
Adjuvant therapy			
Yes	28 (37.8)	73 (35.4)	0.016
No	46 (62.2)	59 (64.6)	
^†^CEA (ng/ml)			
>5	33 (44.6)	56 (42.4)	0.763
≤5	41 (55.4)	76 (57.6)	
Pathological stage			
Stages I-II	42 (56.8)	67 (50.8)	0.408
Stages III-IV	32 (43.2)	65 (49.2)	
Differentiated degree			
High-middle	61 (82.4)	112 (84.8)	0.650
Minor	13 (17.6)	20 (15.2)	
Lymphatic invasion			
Yes	32 (43.2)	67 (50.8)	0.300
No	42 (56.8)	65 (49.2)	
Vascular invasion			
Yes	27 (36.5)	72 (54.5)	0.013
No	47 (63.5)	60 (45.5)	

^†^KPS: ∆SII: the change in the systemic immune-inflammation index; KPS: Karnofsky Performance Status; CEA: carcinoembryonic antigen.

**Table 3:** Univariable and multivariable analysis for overall survival.

Variables	Univariable analysis			Multivariable analysis		
	HR	95% CI	*p*	HR	95% CI	*p*
Age (≤60/>60, years)	1.162	0.693-1.946	0.569			
Gender (male/female)	1.300	0.795-2.126	0.295			
^†^KPS (>80/≤80)	0.841	0.492-1.439	0.527			
Smoking status (yes/no)	1.890	1.028-3.476	0.040			
Localization (rectum/colon)	0.947	0.575-1.560	0.831			
Surgical approach (laparoscopy/open)	0.985	0.601-1.615	0.952			
Adjuvant therapy (no/yes)	0.652	0.398-1.069	0.090			
^†^CEA (≤5/>5, ng/ml)	0.692	0.417-1.147	0.153			
Pathological stage (I–II/III–IV)	0.451	0.270-0.755	0.002	0.442	0.236-0.827	0.011
Differentiated degree (high-middle/minor)	0.476	0.262-0.862	0.014			
Lymphatic invasion (yes/no)	1.934	1.159-3.227	0.012			
Vascular invasion (yes/no)	3.508	1.771-5.279	<0.001	2.182	1.243-3.829	0.007
^†^ *Δ*SII (>127.7/≤127.7)	4.281	2.553-7.719	<0.001	4.301	2.517-7.350	<0.001

^†^KPS: ∆SII: the change in the systemic immune-inflammation index; KPS: Karnofsky Performance Status; CEA: carcinoembryonic antigen.

**Table 4:** Univariable and multivariable analysis for disease-free survival.

Variables	Univariable analysis			Multivariable analysis		
	HR	95% CI	*p*	HR	95% CI	*p*
Age (≤60/>60, years)	1.100	0.682-1.773	0.696			
Gender (male/female)	1.342	0.850-2.119	0.207			
^†^KPS (>80/≤80)	0.785	0.477-1.293	0.343			
Smoking status (yes/no)	1.401	0.832-2.361	0.205			
Localization (rectum/colon)	0.824	0.515-1.318	0.420			
Surgical approach (laparoscopy/open)	0.929	0.588-1.470	0.754			
Adjuvant therapy (no/yes)	0.627	0.395-0.993	0.047	0.415	0.250-0.687	0.001
^†^CEA (≤5/>5, ng/ml)	0.818	0.514-1.302	0.396			
Pathological stage (I–II/III–IV)	0.512	0.321-0.814	0.005			
Differentiated degree (high-middle/minor)	0.624	0.349-1.118	0.113			
Lymphatic invasion (yes/no)	1.583	0.994-2.521	0.053			
Vascular invasion (yes/no)	3.572	2.118-6.024	<0.001	3.305	1.944-5.620	<0.001
^†^ *Δ*SII (>127.7/≤127.7)	4.041	2.514-6.496	<0.001	4.924	2.992-8.102	<0.001

^†^KPS: ∆SII: the change in the systemic immune-inflammation index; KPS: Karnofsky Performance Status; CEA: carcinoembryonic antigen.

## Data Availability

The datasets generated during and analyzed during the current study are available from the corresponding author on reasonable request.
